# Solubility Enhancement of Raloxifene Using Inclusion Complexes and Cogrinding Method

**DOI:** 10.1155/2013/527380

**Published:** 2013-01-29

**Authors:** Payal H. Patil, Veena S. Belgamwar, Pratibha R. Patil, Sanjay J. Surana

**Affiliations:** Department of Pharmaceutics, R. C. Patel Institute of Pharmaceutical Education and Research, Near Karvand Naka, Shirpur, Dhule, Maharashtra 425405, India

## Abstract

The objective of the present work was to enhance the solubility and dissolution of practically water-insoluble drug raloxifene HCl (RLX), for the same two approaches that were used. In the first approach, drug was kneaded with hydroxypropyl-*β*-cyclodextrin (HP*β*CD), and in the second one drug was cogrinded with modified guar gum (MGG). The drug-cyclodextrin complex and drug-MGG cogrind mixtures were characterized by differential scanning calorimetry, X-ray diffraction studies, scanning electron microscopy, and Fourier transform infrared spectroscopy. The solubility and dissolution study reveals that solubility and dissolution rate of RLX remarkably increased in both methods. It was concluded that the prepared inclusion complex showed a remarkable increase in solubility and dissolution of poorly water-soluble drug raloxifene. In the cogrinding mixture, a natural modified gum is used as a surfactant and enhances the solubility and dissolution of RLX without requiring addition of organic solvent or high temperature for its preparation; thus, process is less cumbersome and cost effective. But when both methods were compared; HP*β*CD complexation method showed significant enhancement of drug solubility.

## 1. Introduction

Solubility of a drug is an important property that mainly influences the extent of oral bioavailability. Enhancement of oral bioavailability of poorly water soluble drugs is the most challenging aspect of drug development [1]. Most of the new chemical entities suffer from low bioavailability due to their low aqueous solubility and dissolution. Therefore, it is very important to find appropriate formulation approaches to improve aqueous solubility and bioavailability of poorly aqueous soluble drugs [2].

Raloxifene (marketed as Evista by Eli Lilly and Company) is an oral second generation selective estrogen receptor modulator (SERM) used to prevent osteoporosis in postmenopausal women. It is 2-(4-hydroxyphenyl)-3-({4-[2-(piperidin-1-yl) ethoxy] phenyl} carbonyl)-1-benzothiophen-6-ol that has estrogenic actions on bone and antiestrogenic actions on the uterus and breast. It belongs to class II drug according to biopharmaceutical classification system (BCS), that is, low solubility and high permeability. But raloxifene has very less bioavailability, only 2%, and it would be advantageous to increase the solubility of such molecule. Raloxifene is available in salt form as raloxifene HCl [3]. The drug is poorly absorbed from the gastrointestinal (GI) tract therefore; it is important to enhance aqueous solubility and dissolution rate which may lead to enhancement of bioavailability from its oral solid dosage forms.

In this study, two strategies were used, which were aimed at improving the aqueous solubility. The first one is complexation of drugs with cyclodextrin, and the second one is Cogrinding with natural polymers. Cyclodextrins (CDs) are cyclic macromolecules, obtained by the degradation of starch by *α*-1,4-glucan-glycosyl transferase. They have also been used to improve drug stability, bioavailability, or toxicity profiles. Moreover, chemically modified cyclodextrins have been extensively used. Among the chemically modified cyclodextrins, 2-hydroxypropyl-*β*-cyclodextrin (HP-*β*-CD) deserves special attention due to its favorable physicochemical and biological properties [4]. Kneading method was employed for the preparation of raloxifene HCl with HP*β*CD, and the effect of complexation on the solubility and dissolution rate of raloxifene was evaluated. In the second approach, Cogrinding of raloxifene HCl with modified guar gum was done. Guar gum (GG) is a gum obtained from the ground endosperms of *Cyamopsis tetragonolobus *(Leguminosae family). It is composed of galactan and mannan units combined through glycosidic linkages [5]. The natural polymers have surfactant activity [6] that enhances the solubility and dissolution rate of drug, but high viscosity of these polymers is a limitation for them to be used as carriers for dissolution enhancement [7]; this problem is overcome by heating of the polymers at particular time and temperature condition which reduces the viscosity [6].

The present study was carried out to investigate the inclusion complex of raloxifene HCl and HP*β*CD in the solid state and the RLX-MGG Cogrind mixture using X-ray diffractometry (XRD) differential scanning calorimetry (DSC), Fourier transform infrared spectroscopy (FTIR), and scanning electron microscopy (SEM). The objective of this study was to enhance solubility and dissolution rate of RLX which may lead to enhancement of bioavailability of this drug [1, 8, 9].

## 2. Materials and Methods

### 2.1. Materials

Raloxifene (RLX) was obtained as a gift sample from Zydus Cadila Healthcare Ltd., Ahmedabad, India. HP*β*CD was gifted by Roquette Pharma, France and, Guar gum (GG) was gifted by Lucid Colloids Ltd., Sewari, Mumbai. Methanol and all other reagents used were of analytical grade.

### 2.2. Methods

#### 2.2.1. Kneading Method [8, 10]

*Development of Inclusion Complex of Raloxifene with HPβCD.* As raloxifene HCl is practically insoluble in water, an inclusion complex of the antiosteoporotic raloxifene HCl (RLX) in hydroxypropyl-*β*-cyclodextrin (HP*β*CD) was prepared and characterized. 

*Preparation of RLX-HPβCD Inclusion Complex.* It was prepared by Kneading method. The mixture of RLX and HP*β*CD in 1 : 1 molar ratio was triturated in a mortar with a small volume of water-methanol (1 : 2 v/v) solution. The thick slurry formed was kneaded for 45 min and then dried at 45°C. The dried mass was pulverized and sieved through sieve no. 60. 

#### 2.2.2. Cogrinding Method

*Modification of Polymers [1].* Guar Gum was modified by heating method. Powdered gum was taken in a porcelain bowl and kept in hot air oven at different temperatures and different time intervals. The viscosity and swelling index were studied, which reveals that viscosity decreases as the time and temperature of heating increases but swelling index remains unaffected. It was observed that guar gum produced colour change on heating above 130°C and 120°C more than 2 hrs respectively. Thus, 120°C and 2 hrs conditions were selected for modification of polymers. Finally, modified gum was sieved through mesh no. 100 and stored in airtight container. 

*Characterization of Polymers*

*Swelling Index (SI) [1, 9, 11].* About 1 gm of GG and MGG were accurately weighed and transferred to 100 mL measuring cylinder. The initial volume of powder in measuring cylinder was noted which is denoted as *X*_0_. Distilled water was added in measuring cylinder up to 100 mL mark, shaken gently, and cylinder was kept aside for 24 hrs. The final volume occupied by polymers was noted after 24 hrs, which is denoted as *X*_*t*_. Swelling index was calculated according to the following equation:

(1) $$\begin{array}{c} \text{SI}=\frac{X_{t}-X_{0}}{X_{0\;}}\times 100. \end{array}$$

*Viscosity Measurement [9]*. Viscosity of GG and MGG gums was determined by using Brookfield DV–E viscometer (Brookfield engineering laboratory) at 37°C and 50 rpm. 1% (w/v) solution was prepared in distilled water and used for measuring the viscosity.

*Preparation of Cogrind Mixture [1].* Cogrind mixtures of drug and modified gum were prepared in different ratio such as 1 : 1 to 1 : 9. The ratio was optimized by using solubility data. The sample of drug and gum in 1 : 1 w/w ratio was Cogrinded for 25 min, in ceramic mortar and sieved through mesh no. 100. The same method was applied for all ratios of drug with polymer. The Cogrind mixture of RLX with MGG and GG denoted as RLX-MGG and RLX-GG, respectively.

*Ratio Optimization (Drug : Polymer).* Samples were placed in 10 mL solvent (pH 7 phosphate buffer) in teflon facing screw capped vial and kept at equilibrium for a period of 24 hrs on orbital shaking incubator (Remi Instruments Ltd.) at 37 ± 0.5°C and 50 rpm. The contents of vials were filtered through 0.2 micron filter and analyzed by UV-Visible spectrophotometer (UV 1601, Shimadzu) at 287 nm. As shown in Table 3, the solubility increases as the gum concentration increases, the optimized ratio was found to be 1 : 8 w/w as further increase in ratio to 1 : 9 w/w showed no significant increase in solubility of drug. Cogrinding mixtures of RLX with MGG and GG were prepared in 1 : 8 w/w ratio.

#### 2.2.3. Characterization of HP*β*CD Inclusion Complex and Cogrind Mixture [1, 9]

1. *Differential Scanning Calorimetry (DSC).* DSC studies of raloxifene HCl, HP*β*CD, MGG, RLX-HP*β*CD inclusion complex, and RLX-MGG Cogrind mixture were performed using differential scanning calorimeter (Mettler Toledo DSC 1 Star System, Zurich, Switzerland) at heating rate of 10°C/min from 40 to 340°C in nitrogen atmosphere.
2. *X-Ray Diffractometry (XRD).* Powder XRD patterns of raloxifene HCl, HP*β*CD, MGG, RLX-HP*β*CD inclusion complex, and RLX-MGG Cogrind mixture were recorded using diffractogram (Bruker AXS, D8 Advance, Germany) and Cu-K*α* radiation. Diffractogram was run at a scanning speed of 2°/min and a chart speed of 2°/2 cm per 2*θ*.
3. *Fourier Transform Infrared Spectroscopy (FT-IR).* Raloxifene HCl, HP*β*CD, MGG, RLX-HP*β*CD inclusion complex, and RLX-MGG Cogrind mixture were mixed separately with IR grade KBr in the ratio of 1 : 100, and corresponding pellets were prepared by applying 10 metric ton of pressure in hydraulic press. The pellets were then scanned over a wave range of 4000–400 cm^−1^ in FTIR instrument (8400 S Shimadzu).
4. *Scanning Electron Microscopy (SEM).* The SEM photographs of raloxifene HCl, RLX-HP*β*CD inclusion complex, and RLX-MGG Cogrind mixture were obtained by scanning electron microscope (JSM 6390LV, JEOL Model, Japan) with 10 kV accelerating voltage.

*Phase Solubility Studies of Inclusion Complex [10, 12].* The phase solubility technique permits the evaluation of the affinity between HP*β*CD and raloxifene in water. Phase solubility studies were performed according to the method reported by Higuchi and Connors [13]. As given in Table 1, raloxifene was taken into vials in an excess amount, and 20 mL of distilled water was added, containing various concentration of HP*β*CD (10–40 mmol). These vials were sealed and shaken at 20°C for 4 days. This period was considered sufficient to reach equilibrium. Subsequently, the aliquots were withdrawn, using a syringe, and samples were filtered through 0.2 micron filter and appropriately diluted. A portion of the sample was analyzed by UV spectrophotometer (UV 1601, Shimadzu) at 287 nm against blank prepared in the same concentration of HP*β*CD in water so as to cancel any absorbance that may be exhibited by the HP*β*CD. The solubility experiments were conducted in triplicate. The apparent stability constant (*K*_*c*_) of complexes was calculated from the phase solubility diagram using the following equation:

(2) $$\begin{array}{c} K_{c}=\frac{\text{Slope}}{S_{0}\left(1-\text{slope}\right)}. \end{array}$$

The slope obtained from the initial straight line portion of the plot of raloxifene concentration against HP*β*CD concentration, and *S*_0_ is the equilibrium solubility of raloxifene in water.

*Solubility Study of Cogrind Mixture [1, 9].* The solubility of RLX, RLX-GG and RLX-MGG were determined in distilled water, 1.2 pH HCl buffer, and 7.0 pH buffer. The solubility of drug and Cogrinding mixtures were determined by taking an excess amount 30 mg of drug and the Cogrind mixture equivalent to 30 mg of drug, was added in 10 mL of the previous solvents, in teflon facing screw capped vials. The samples were kept at equilibrium for a period of 48 hrs on orbital shaking incubator at 37 ± 0.5°C and 50 rpm. The contents of vials were filtered through 0.2 micron filter and analyzed by UV-Visible spectrophotometer (UV 1601, Shimadzu) at 287 nm.

#### 2.2.4. *In Vitro* Dissolution Rate Study [9]

Dissolution rates from RLX, RLX-HP*β*CD inclusion complex, and RLX-MGG Cogrind mixture were determined in 900 mL of pH 6.8 phosphate buffer at 37 ± 0.5°C with a stirrer rotation speed of 75 rpm using the USP dissolution test apparatus type II (paddle type) (TDT 08L-ELECTROLAB, Mumbai, India). RLX-HP*β*CD inclusion complex and RLX-MGG Cogrind mixture were taken equivalent to 100 mg of RLX. An aliquot of 5 mL of sample was withdrawn at 5, 10, 15, 30, 45, 60, 90, 105, and 120 min with a pipette. The samples were filtered through 0.2 micron filter, suitably diluted and assayed spectrophotometrically at 287 nm. Each dissolution rate test was repeated three times. As a model independent approach, dissolution efficiency (DE) was employed to evaluate the dissolution rate of RLX. DE is defined as the area under the dissolution curve up to the time *t*, expressed as a percentage of the area of the rectangle described by 100% dissolution in the same time. DE_60_ and DE_120_ were calculated from the dissolution data and used for comparison.

#### 2.2.5. Statistical Evaluation

All results are expressed as mean ± S.D. Differences between the two related parameters were considered statistically significant for *P* values for less than 0.05. Drug to polymer *optimization* ratio, solubility determination, and dissolution efficiency results were analyzed by applying one way ANOVA test.

## 3. Results and Discussion

### 3.1. Development of Inclusion Complex of Raloxifene and HP*β*CD

Cyclodextrin (CD) has a hydrophobic central cavity and hydrophilic outer surface and can encapsulate model substrates to form host-guest complexes or supramolecular species. This usually enhances drug solubility in aqueous solution and affects the chemical characteristics of the encapsulated drug. HP*β*CD is a hydroxyalkylated-*β*-cyclodextrin derivative that combines relatively high water solubility with low toxicity and satisfactory inclusion ability. The binding behavior of hydroxypropyl-*β*-cyclodextrin with RLX and the solubilization effect of HP*β*CD toward RLX may provide a useful approach to produce a novel RLX formulation with improved bioavailability. 

### 3.2. Development of Cogrind Mixture of Raloxifene and Modified Guar Gum

The natural polymers are mainly evaluated in industry for their new applications. Due to the less toxic effect and low production cost, these polymers mainly used as drug carrier in pharmaceutical industry. Guar gum has surfactant activity [6], which reduces the contact angle and increases wetting of drug particles, thus enhances solubilization and dissolution of drug particles. This gum has limitation as dissolution enhancing carrier due to their high viscosity. These polymers produce gel layer on the hydrated surfaces which prevents the drug release during drug dissolution and reduced the dissolution [14]. It is reported that the swelling of polymers influences improvement of dissolution rate of poorly aqueous soluble drugs [15]. Therefore, it is useful to modify the gum in such a way that its swelling ability remains the same and decreases the viscosity.

#### 3.2.1. Viscosity and Swelling Index Measurement

The results of swelling index and viscosity of polymers are given in Table 2. The result indicates that the viscosity of MGG is lower than that of the GG, and swelling index of MGG was not reduced significantly than the GG. Because of swelling nature of the carrier, the extensive surface was increased during the dissolution and thus dissolution rate of drug was enhanced [16].

### 3.3. Characterization of Inclusion Complex and Cogrind Mixture

#### 3.3.1. Differential Scanning Calorimetry (DSC) Study 

**Figure 1:**
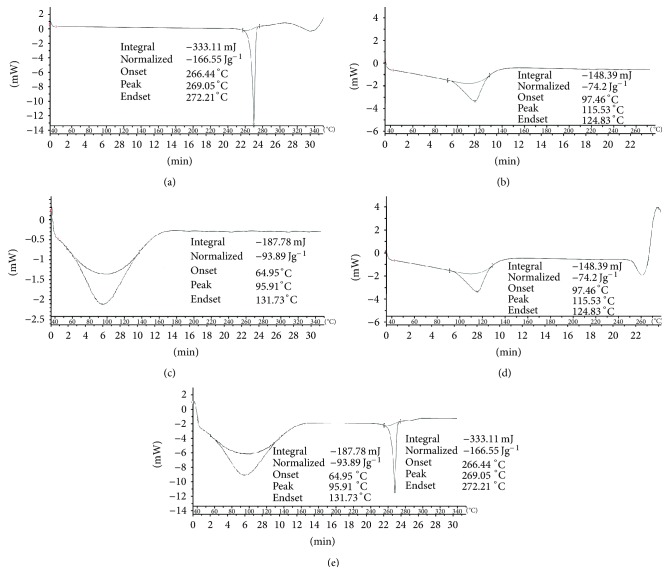
DSC thermogram of (a) raloxifene HCl, (b) HP*β*CD, (c) modified guar gum, (d) raloxifene-HP*β*CD inclusion complex and (e) raloxifene-MGG Cogrind mixture.

As shown in Figure 1, the thermograms of the RLX, HP*β*CD, and MGG showed respective endothermic peaks at 266.44°C, 97.46°C and 64.95°C corresponding to their melting points. In the thermogram of RLX-HP*β*CD complex, the peak of drug disappeared indicating the complexation of RLX with cyclodextrin, whereas in DSC spectra of RLX-MGG Cogrind mixture, the peak of drug was observed, but the intensity was reduced suggesting the conversion of raloxifene hydrochloride from crystalline form to amorphous form.

#### 3.3.2. X-Ray Diffraction Study 

**Figure 2:**
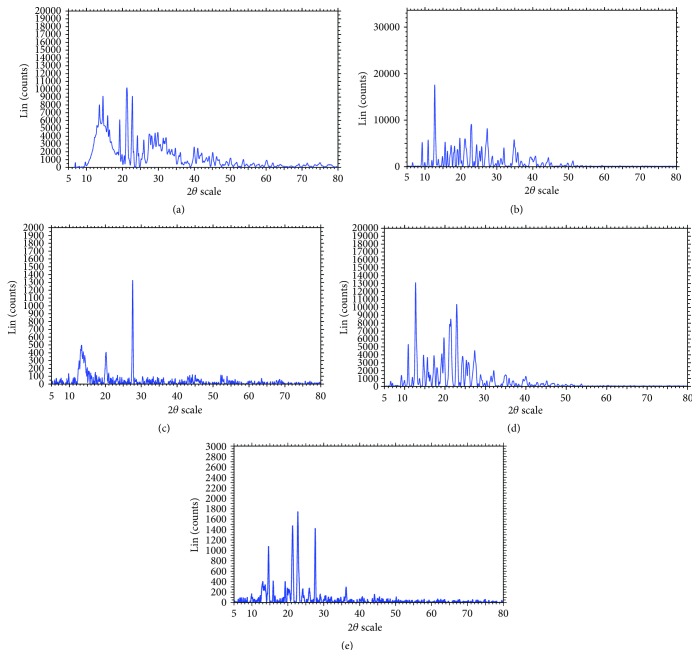
PXRD of (a) raloxifene HCl, (b) HP*β*CD, (c) modified guar gum, (d) raloxifene-HP*β*CD inclusion complex and (e) raloxifene-MGG Cogrind mixture.

As shown in Figure 2, the X-ray diffraction patterns were recorded for pure RLX, HP*β*CD, MGG, RLX-HP*β*CD inclusion complex, and RLX-MGG Cogrind mixture. PXRD studies were performed in conjunction with DSC to verify the reduction of crystallinity of RLX. Diffraction spectrum of drug sample showed distinct peaks at 2*θ* of 12.812°, 14.47°, 15.784°, 19.153°, 22.682°, and 25.876°. All these peaks, though of relatively lesser intensity, were observed to be in RLX-HP*β*CD complex and RLX-MGG Cogrind mixture. It was thus concluded that the drug was converted from crystalline to amorphous state.

#### 3.3.3. Fourier Transform Infrared Spectroscopy (FT-IR)

**Figure 3:**
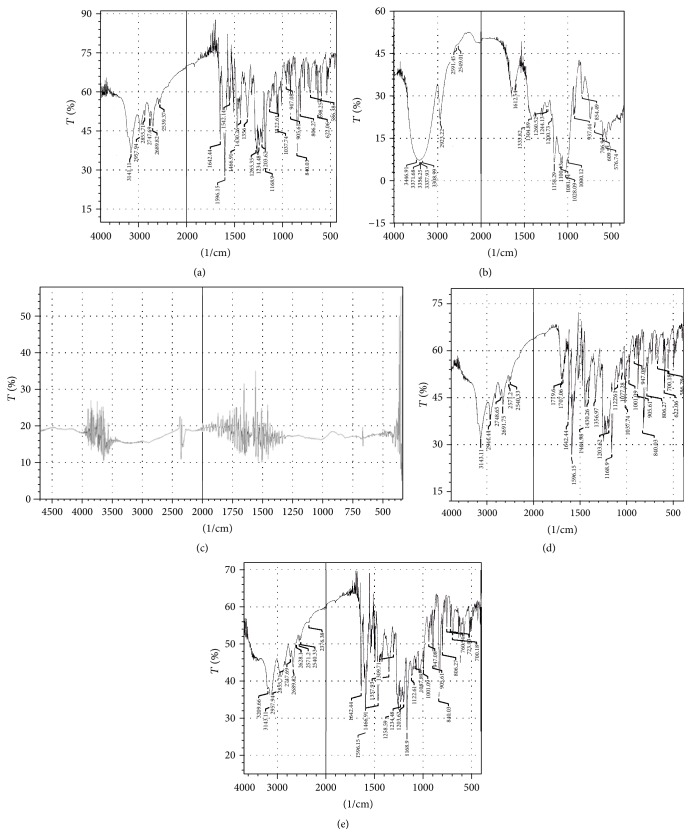
FTIR spectra of (a) raloxifene HCl, (b) HP*β*CD, (c) modified guar gum, (d) raloxifene-HP*β*CD inclusion complex and (e) raloxifene-MGG Cogrind mixture.

As shown in Figure 3, FT-IR spectra of RLX exhibited characteristic peaks at 1,642.44 (C=O stretching), 1,596.15 (–C–O–C– stretching), 1,466.91 (–S–benzothiofuran), and 905.61 cm^−1^ (benzene ring). They were well preserved in the RLX-HP*β*CD complex and RLX-MGG Cogrind mixture. These results indicate that no interaction occurred between drug and excipients.

#### 3.3.4. Scanning Electron Microscopy (SEM)

**Figure 4:**
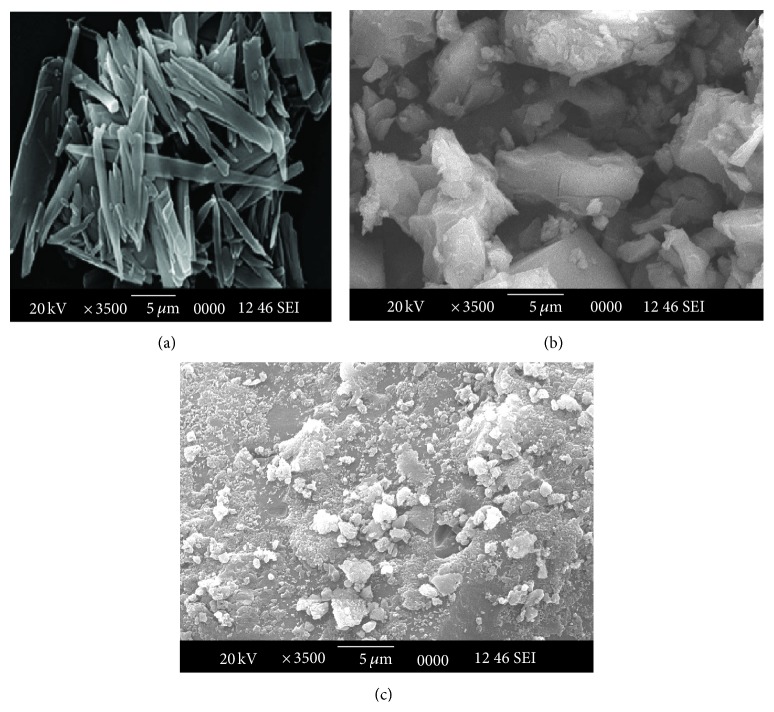
SEM of (a) raloxifene, (b) raloxifene-HP*β*CD inclusion complex and(c) raloxifene-MGG Cogrind mixture.

As shown in Figure 4, the scanning electron microscopy photomicrographs of RLX shown in Figure 4 show the longer crystals with very specific morphology, whereas for RLX-HP*β*CD complex and RLX-MGG Cogrind mixture, a decrease in crystallinity due to formation of drug-cyclodextrin complex and molecular dispersion of RLX in the polymer matrix (MGG) was observed, respectively.

#### 3.3.5. Solubility Study

**Figure 5:**
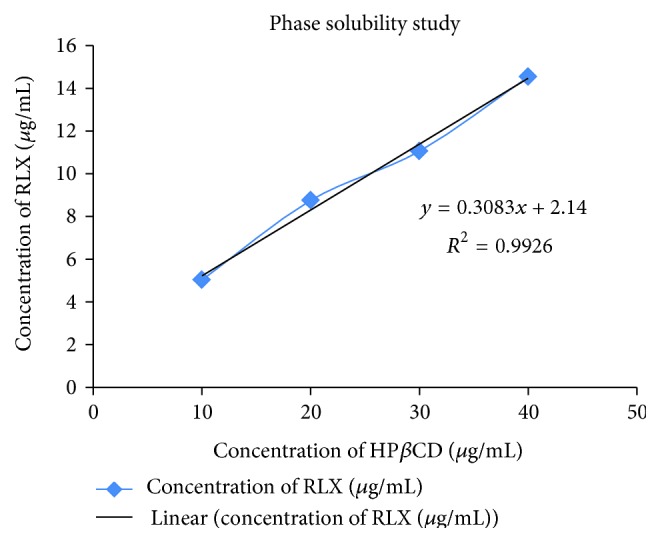
Phase solubility diagram of raloxifene.

*Phase Solubility Study of Inclusion Complex.* The phase solubility diagram for the complex formation between RLX and HP*β*CD is shown in Figure 5. The aqueous solubility of RLX increased linearly with a slope 0.3083 (*r* ^2^ = 0.9926) as a function of HP*β*CD concentration. The apparent solubility constant *K*_*c*_, obtained from the slope of the linear phase solubility diagram was found to be 4.5949 mol^−1^.

*Solubility Study of Cogrind Mixture.* Solubility data for RLX, RLX-GG and RLX-MGG in different solvents are given in Table 4. ANOVA (*P* < 0.05) performed on solubility parameter demonstrated significant difference between solubility of RLX, RLX-GG and RLX-MGG Cogrinding mixtures. Cogrinding mixture of RLX-MGG showed slight better results than RLX-GG; therefore RLX-MGG was selected for further studies. But there was significant difference between solubility of RLX-HP*β*CD inclusion complex and Cogrinding mixtures. 

### 3.4. *In Vitro* Dissolution Rate Study 

**Figure 6:**
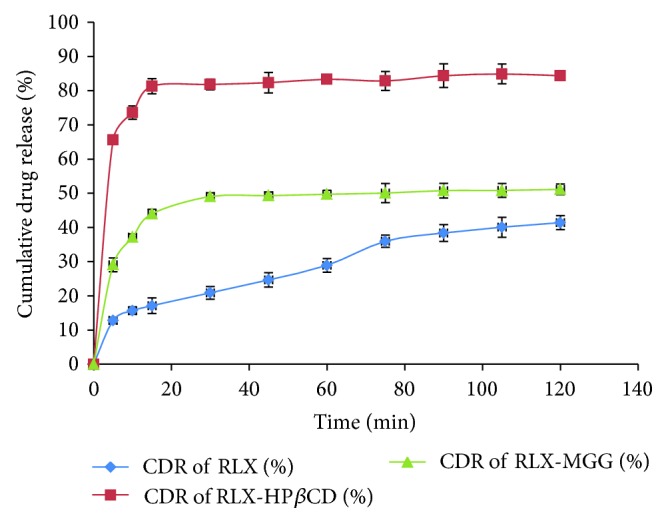
Dissolution profile of RLX, RLX-HP*β*CD inclusion complex, and RLX-MGG Cogrind mixture.

Figure 6 represents *in vitro* dissolution profiles of RLX, RLX-HP*β*CD inclusion complex, and RLX-MGG Cogrind mixture.  Table 5 summarizes % drug release from the RLX-HP*β*CD inclusion complex and RLX-MGG Cogrind mixture at 60 and 120 min. From Table 5, we can conclude that maximum enhancement in dissolution rate up to 84.47% ± 0.84% is shown by RLX-HP*β*CD inclusion complex as compared to that of 51.30 ± 0.57% by RLX-MGG Cogrind mixture at 120 min. The results of the statistical analysis (ANOVA) suggest significant enhancement of dissolution rate of RLX from RLX-HP*β*CD inclusion complex at all the time points (*P* < 0.05) when compared with plain RLX and RLX-MGG Cogrind mixture.

## 4. Conclusion

The molecular structure of cyclodextrin creates a bucket-like cavity that can function to complex with drug or functional groups on drug. The investigation suggests from phase solubility study and dissolution rate profile of the inclusion complex that the solubility and dissolution rate of raloxifene increases significantly due to HP*β*CD. Whereas, the Cogrinding method enhances the solubility of RLX by converting it to amorphous form, reducing the particle size and increasing wettability. The optimum ratio for Cogrinding mixture was found to be 1 : 8 which shows higher solubility. Moreover, this natural polymer like guar gum has advantage over other synthetic polymers as these polymers are biocompatible, biodegradable, and having low cost.

Hence, from practical point of view, Cogrinding method appeared easier and was considered as the most convenient method. But when both these techniques were compared the inclusion complex method showed better results as compared to those of the other methods and thus was found to be more effective than Cogrinding method.

## Figures and Tables

**Table 1:** Phase solubility study of raloxifene HCl in distilled water.

Sr. no.	Formulation code	A1	A2	A3	A4
1	Drug (mg)	20	20	20	20
2	HP*β*CD (mg)	20	40	60	80
3	Distilled Water (mL)	20	20	20	20

**Table 2:** Characterization of guar gum and modified guar gum. All values are mean ± S.D, *n* = 3.

Polymer	Viscosity (cP)	Swelling index (%)
Guar gum	323 ± 18.52	2905.46 ± 6.01
Modified guar gum	197 ± 10.43	2700.69 ± 10.13

**Table 3:** Optimization ratio of RLX with guar gum and modified guar gum. All values are mean ± S.D, *n* = 3.

Ratio	Concentration (mg/mL)	
	RLX-GG	RLX-MGG
1 : 1	1.11 ± 0.041∗	0.87 ± 0.065∗
1 : 2	1.33 ± 0.005∗	1.09 ± 0.004∗
1 : 3	1.40 ± 0.109∗	1.11 ± 0.010∗
1 : 4	1.46 ± 0.017∗	2.13 ± 0.011∗
1 : 5	1.63 ± 0.012∗	2.30 ± 0.031∗
1 : 6	2.01 ± 0.015∗	2.41 ± 0.013∗
1 : 7	2.18 ± 0.056∗	3.54 ± 0.011∗
1 : 8	2.32 ± 0.015∗	3.69 ± 0.012∗
1 : 9	2.35 ± 0.007	3.67 ± 0.023

∗Significant (*P* value < 0.05).

**Table 4:** Solubility of RLX and cogrind mixtures in different solvent at 37 ± 0.5°C after 48 hrs. All values are mean ± S.D, *n* = 3.

Sample	Medium		
	Water (mg/mL)	pH 1.2 HCl buffer (mg/mL)	pH 7 phosphate buffer (mg/mL)
RLX	0.097 ± 0.105	0.082 ± 0.079	0.076 ± 0.023
RLX-GG	2.090 ± 0.002∗	2.037 ± 0.160∗	2.610 ± 0.020∗
RLX-MGG	3.570 ± 0.27∗	3.173 ± 0.030∗	3.870 ± 0.360∗

∗Significant (*P* value < 0.05).

**Table 5:** Dissolution efficiency of RLX, RLX-HP*β*CD inclusion complex, and RLX-MGG cogrind mixture. All values are mean ± S.D, *n* = 3.

Sample	Dissolution efficiency	
	DE_60_	DE_120_
Raloxifene	28.92 ± 1.98∗	41.41 ± 2.04
RLX-HP*β*CD inclusion complex	83.34 ± 1.14∗	84.47 ± 0.84∗
RLX-MGG cogrind mixture	49.70 ± 1.18∗	51.30 ± 1.57∗

∗Significant (*P* value < 0.05).
